# Breathing SPACE—a practical approach to the breathless patient

**DOI:** 10.1038/s41533-016-0006-6

**Published:** 2017-01-30

**Authors:** Nicholas S. Hopkinson, Noel Baxter, on behalf of the London Respiratory Network

**Affiliations:** 1 NIHR Respiratory Biomedical Research Unit at Royal Brompton and Harefield NHS Foundation Trust and Imperial College London, London, UK; 2 Southwark Clinical Commissioning Group, London, UK

## Abstract

Breathlessness is a common symptom that may have multiple causes in any one individual and causes that may change over time. Breathlessness campaigns encourage people to see their General Practitioner if they are unduly breathless. Members of the London Respiratory Network collaborated to develop a tool that would encourage a holistic approach to breathlessness, which was applicable both at the time of diagnosis and during ongoing management. This has led to the development of the aide memoire “Breathing SPACE”, which encompasses five key themes—smoking, pulmonary disease, anxiety/psychosocial factors, cardiac disease, and exercise/fitness. A particular concern was to ensure that high-value interventions (smoking cessation and exercise interventions) are prioritised across the life-course and throughout the course of disease management. The approach is relevant both to well people and in those with an underling diagnosis or diagnoses. The inclusion of anxiety draws attention to the importance of mental health issues. Parity of esteem requires the physical health problems of people with mental illness to be addressed. The SPACE mnemonic also addresses the problem of underdiagnosis of heart disease in people with lung disease and vice versa, as well as the systematic undertreatment of these conditions where they do co-occur.

## Introduction

Breathlessness is one of the most common symptoms causing people to interact with the health-care system, present in about 10% of all adults and 30% of older people.^1,2^ It can be frightening and disabling, but many people ignore or normalise the symptom and fail to act on it. This can delay diagnosis and effective interventions, both medical and “lifestyle”. For example, data on patients with chronic obstructive pulmonary disease (COPD) show clearly that, for many individuals, opportunities are missed to make a diagnosis in the years prior to the condition finally being recognised.^3,4^ Multimorbidity is common^5^ and breathlessness in any individual is likely to be multifactorial.^6^ The London Respiratory Network wished to develop an aide memoire to facilitate a systematic approach to this. Consensus discussions identified smoking, pulmonary disease, anxiety, cardiac disease, and exercise level/unfitness as important and often interacting contributors to breathlessness, which can be encompassed by the acronym “Breathing SPACE” (Table 1). These items should be considered in any breathless patient, both during the diagnostic process and their subsequent management, though they may not all be addressed in a single consultation. The approach is intended to be relevant both to health-care practitioners and to commissioners of health-care services.

**Table 1:** The Breathing SPACE framework

**S**moking cessation	Ask about smoking, advise to quit, offer assistance to quit. Adopt the CO4 approach^7^:
	1. The right **CO**nversation, with every patient and staff member who smokes, that gives them a chance to quit, referring for support if necessary
	2. Offer routine exhaled carbon monoxide (**CO**) monitoring: “Would you like to know your level?”
	3. **CO**de smoking cessation interventions and include smoking history in death certification
	4. **CO**mmission services where smoking cessation behaviours are incentivised systematically
**P**ulmonary disease	Ask about symptoms—cough, sputum, variability, nocturnal symptoms, chest discomfort, haemoptysis
	Investigations—rapid access to quality assured spirometry
	Pulse oximetry
	Prioritise high-value interventions—smoking cessation, pulmonary rehabilitation, flu vaccination
	Ensure that inhaled medications are both prescribed and used appropriately
**A**nxiety	Psychosocial factors contribute to symptoms in cardiorespiratory disease. Anxiety may present with specific features of hyperventilation/dysfunctional breathing syndrome, including paraesthesia and “air hunger”
	Parity of esteem—address the physical health of people with mental health issues
	Smoking cessation interventions are effective and safe to use in people with mental health problems
	Peer support, e.g., BLF Breathe Easy groups
**C**ardiac disease	Ask about risk factors (smoking, hypertension, diabetes, ischaemic heart disease?)
	Abnormal pulse, pulmonary crepitations, oedema, cardiac murmurs
	Cardiac complications of respiratory disease—pulmonary hypertension, sleep-disordered breathing
	Investigations—consider ECG, BNP, echocardiogram
	Refer patients with heart disease who feel limited by their symptoms for exercise rehabilitation
**E**xercise level and	Ask about exercise level “Do you take any regular exercise?”
fitness	Give brief advice to increase physical activity levels^8^
	Reassure and encourage: “It’s not harmful to make yourself breathless”
	Refer patients with lung or heart disease who feel limited by their symptoms for exercise rehabilitation
	Obesity—identify this explicitly as a contributor to breathlessness
	Signpost opportunities to participate in exercise (e.g., Park Run, Couch to 5K). Pedometer-based interventions with a step count goal are effective^9,10^

**Fig. 1:**
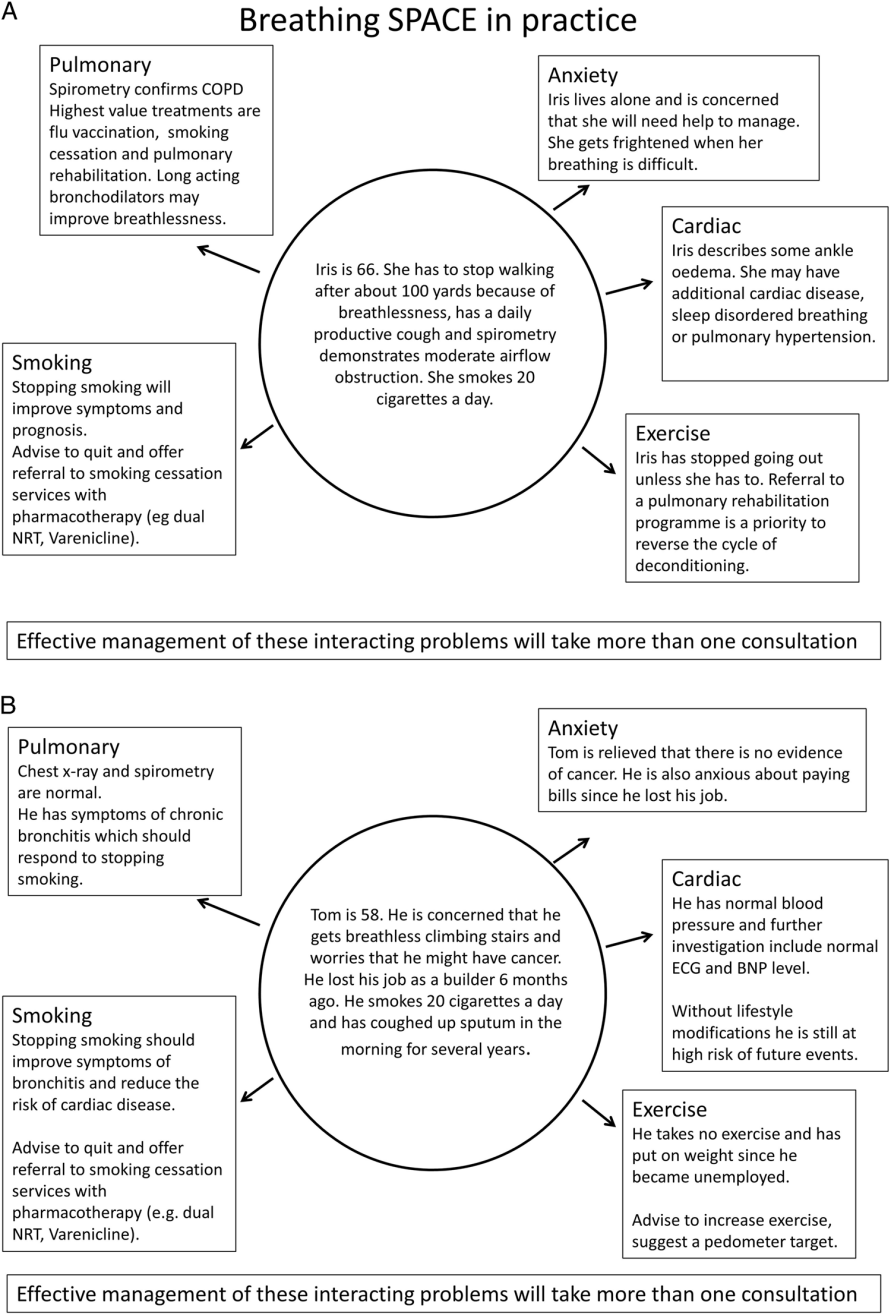
Application of Breathing SPACE in a typical patient with COPD (**a**) and in a patient where the main issue is deconditioning and lifestyle(**b**). In a typical patient with COPD, all five of the SPACE elements may be in play with attention required to **S**moking cessation, treatments directly for the **P**ulmonary condition (vaccination, rehabilitation, inhaled medication), **A**nxiety about going outside for fear of being seen to be breathless, ensuring medication for a **C**ardiac co-morbidity is optimised, and a need to continue to consider increase **E**xercise levels following pulmonary rehabilitation and behaviour change

The application of this approach in two typical patients, one with COPD and one where deconditioning is the main issue, is illustrated in Fig. 1.

## Smoking

Smoking is a leading preventable *cause* of ill health, but smoking cessation is also one of the most effective *treatment*s for many long-term conditions.^11–14^ Ask breathless patients about smoking history, encourage smokers to quit and offer assistance to overcome their tobacco dependence. Brief smoking cessation advice is an effective intervention,^15^ but counselling support combined with pharmacotherapy is more so.^16^ For example, the cost per quality adjusted life year of combined smoking cessation counselling and pharmacotherapy in COPD is only €2400, compared with €8200 for intensive counselling and €16,900 for brief advice.^17^ Providing evidence-based smoking cessation for inpatients who smoke has been shown not only to significantly reduce readmissions at 30 days (by about half), but also to significantly reduce mortality at 1 year (again by about a half).^13^ Smoking is highly prevalent in socioeconomically deprived groups and in those with mental health problems,^18^ and support to these groups should be prioritised. The National Centre for Smoking Cessation and Training website (www.ncsct.co.uk/) is a useful resource, including online training.

## Pulmonary disease

**Fig. 2:**
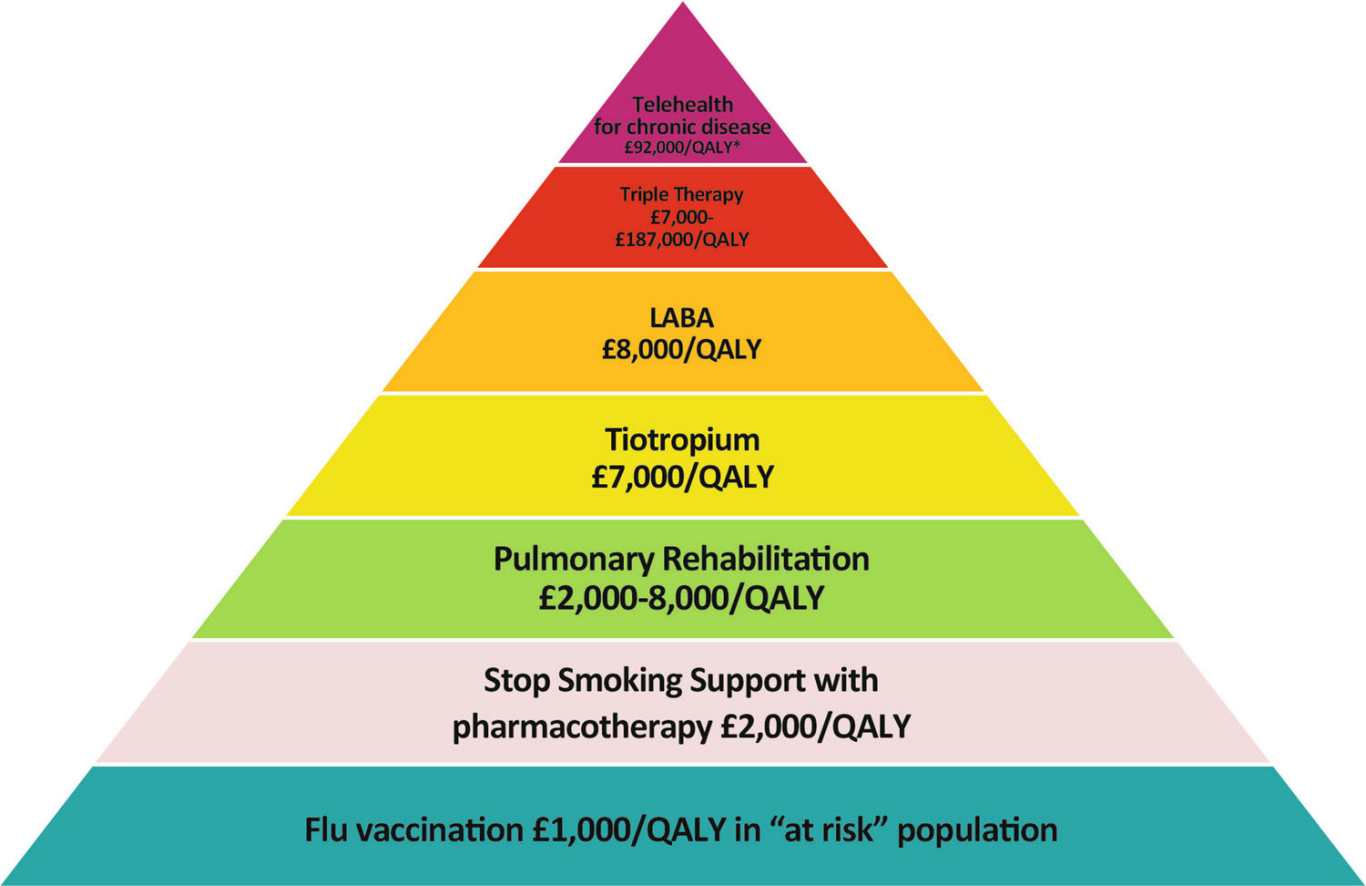
The pyramid of value for COPD interventions.The pyramid of value for COPD interventions developed by the London Respiratory Network with The London School of Economics (reproduced from^22^) gives estimates of cost per quality-adjusted life year gained. *LABA* long-acting β2 agonist, *QALY* quality-adjusted life year

Breathlessness is a characteristic manifestation of pulmonary diseases such as COPD and asthma. Suggestive features may trigger a referral, or investigations such as a chest X-ray. Perform quality-assured spirometry promptly on any patient with symptoms of long-term breathlessness. This can be diagnostic for airflow obstruction and the demonstration of abnormal lung function provides an additional incentive for smokers to quit.^19^ Asthma is by definition variable so normal spirometry does not rule it out. Timely diagnosis of COPD also has good value—low diagnostic rates of COPD are associated with higher disease-specific costs^20^ and prevalence of undiagnosed COPD is strongly associated with rates of hospital admission for acute exacerbations.^21^ Prioritise high-value interventions, including flu vaccination, smoking cessation, and pulmonary rehabilitation, which have significantly lower costs per QALY than pharmacotherapy (Fig. 2).^22^

## Anxiety

Anxiety is taken here to represent a range of psychosocial problems. Anxiety disorders can present with breathlessness as a primary symptom. Alternatively, anxiety can aggravate the symptoms of a long-term condition such as COPD or heart failure.^23–25^ A key element of “parity of esteem” for mental health is that the physical health problems of people with mental health conditions are no longer neglected. Smoking rates are high in people with mental illness and contribute considerably to reduced life expectancy.^18^ Smoking cessation interventions are safe and likely to be as effective in people with a mental health diagnosis as those without.^26–28^

Pulmonary and cardiac rehabilitation improve mental as well as physical components of health status.^29,30^ Although patients with mental health problems benefit from rehabilitation, they may be less likely to attend or complete programmes^31^—identifying people who need additional support and encouragement to attend and continue rehabilitation is important. For some individuals, participation in peer support activities like British Lung Foundation Breathe Easy Groups may be beneficial. Consider referral for psychological therapy; cognitive-behavioural therapy is efficacious in people with respiratory disease and anxiety.^32^

## Cardiac causes

A cardiac cause of breathlessness is suggested by a history of hypertension, diabetes, hypercholesterolaemia or ischaemic heart disease, and by physical findings such as lung crepitations, the presence of oedema, tachycardia, cardiac murmurs, or atrial fibrillation. A normal electrocardiogram (ECG) reduces the likelihood of cardiac disease but consider investigations such as blood natriuretic peptide (BNP) and echocardiography if there is clinical suspicion.^33^

Lung disease is also common in people with heart disease,^5^ so the possibility of a dual diagnosis should be considered. For example, 15–24% of people with COPD also have ischaemic heart disease^5^ and one-third of people with atrial fibrillation have a diagnosis of asthma or COPD.^34^ Evidence suggests systematic undertreatment of patients with COPD following myocardial infarction; they are less likely to receive beta blockers, although these can be safely administered.^35^

Hyperinflation caused by airflow obstruction restricts cardiac output^36^ and the “cardiac” aspects of lung diagnoses should be considered. In patients with COPD, low oxygen saturations, especially if they are disproportionate to the degree of airflow obstruction, may represent pulmonary hypertension that can lead to right-sided heart failure. Consider sleep-disordered breathing with nocturnal hypoxia in individuals with a high body mass index (BMI), oedema, or a history of type II respiratory failure. Ask about daytime somnolence, morning headaches, and confusion.

## Exercise level and fitness

Many people find that their exercise capacity decreases as they get older and attribute this to aging when it is in fact a consequence of physical inactivity and deconditioning.^37^ Excluding the presence of a specific pulmonary, cardiac, or psychological pathology is likely to be the priority in a patient presenting with breathlessness, but even having done this there is evidence that brief interventions to increase physical activity in apparently healthy people are cost-effective.^8,38^

People aging with a sedentary lifestyle are at increased risk of developing many health conditions, including hypertension, obesity, diabetes, depression, osteoporosis, frailty, and falls. Adoption of walking and cycling, as well as improving the fitness of individuals and reducing their risk of future disease, also has other benefits.^39^ It reduces the emission of pollutants (particulates, NO_2_), which increase the risk of developing conditions like asthma and worsen outcomes in people who already have cardiorespiratory disease. The reduction in carbon emissions from adopting active transport also brings a climate change benefit.

Exercise rehabilitation is known to be an effective intervention in cardiorespiratory disease^29,40–42^ and pulmonary rehabilitation is one of the highest value interventions in COPD.^22^ Of note, activity levels are reduced even in early COPD^43^ and inactivity is associated with more rapid decline in lung function in healthy populations and with more rapid progression of COPD.^44^ Although it has not been subjected to prospective randomised controlled trials, these observations do suggest that, in people diagnosed at an early stage, robust encouragement to increase activity level with a clear explanation of the benefits of doing so may alter the way that their lung condition progresses as they age. Of equal importance, increasing physical activity should also limit the development and impact of multi-morbidities—including hypertension, diabetes, osteoporosis, depression, and cognitive dysfunction—that are known to accumulate through the impact of progressive sedentarism.^45^ A person who understands the need to push against the limits of their breathlessness as part of their self-management of a long-term condition, and believes in their capacity to do so, could be expected to maintain their performance better than someone who is slowly withdrawing from activities in the face of unexplained breathlessness due to progressive, undiagnosed, cardiorespiratory disease. A key message for patients is that it is not harmful to make yourself breathless. Pedometer-based interventions with a step count goal are effective.^9,10^

## A coordinated approach—multimorbidity

Multimorbidity is the norm rather than the exception; only 20% of people with COPD have it as their only long-term condition.^5^ Exercise and smoking cessation will benefit other conditions such as hypertension and diabetes, as well as cardiac and pulmonary disease. Anaemia may present with breathlessness and is potentially reversible. Where a referral is made it should ideally be to a service that can function in a way that is symptom specific, dealing with “breathlessness”, rather than to one that can only investigate from a cardiac or respiratory perspective with the patient requiring a second referral if one or other condition is excluded. Table 2 includes recommendations for commissioners, and further recommendations are available in this European Respiratory Society Monograph.^46^

**Table 2:** Breathing SPACE—challenges for commissioners of health and social care

Invest in a population-based approach, describing aims, objectives, and criteria to evaluate the impact of a breathlessness system. Adapt and develop local provision to reduce waste and increase value: know your neighbourhood and local services (tobacco dependence, obesity and physical activity services will vary considerably in the United Kingdom and internationally).
1. Define the scope of the breathlessness system.
2. Define the population to be served, which may include sub-populations or segments at different levels of complexity and activation requiring different services. It may include people with complex needs, such as homeless people, who are known to many service providers, including General Practitioner, ambulance services, emergency departments, and respiratory departments, who would benefit from better care coordination to improve their breathlessness.
3. Reach agreement on the aim and objectives of the services provided by the system, also considering options for disinvestment.
4. For each objective, agree one or more criteria by which the performance of the service would be assessed.
5. For each of the criteria, identify levels of performance that can be used as quality standards, based on the data locally available.
6. Identify all the resources used in the system, thus creating a breathlessness budget, including clinical staff, equipment, diagnostic tests, hospital beds, prescribing budgets, estates, and administration.
7. Identify who needs to be engaged in a clinical network that will provide collective leadership for the system and be accountable for its performance.
8. Produce a breathlessness system specification, which can be used for contractual arrangements between providers and payers.
9. Agree upon an evaluation framework to assess the impact of the breathlessness system.
Commission smoking cessation services—local authority and clinical commissioners should work together to consider where smoking cessation services will have the greatest impact on their joint outcome measures. These are extremely of high value compared with other health interventions and must be protected.
Nine million people still smoke in the United Kingdom—increasingly concentrated in harder to reach groups. Incorporate smoking cessation into care pathways for mental health services, people admitted to hospital, and the homeless.
Commission pathways for breathlessness diagnostics that can address both cardiac and respiratory causes from general practice to specialist units. The IMPRESS decision support tool provides an outline of the key decision-making processes in these clinical encounters (https://www.networks.nhs.uk/nhs-networks/impress-improving-and-integrating-respiratory/news/impress-breathlessness-resources)
Systems should address the under-diagnosis and treatment of cardiac disease in people with respiratory diagnoses, and the under-diagnosis and treatment of respiratory disease in people with cardiac diagnoses
Ensure that palliative care services are also available for patients with breathlessness due to non-malignant conditions
Parity of esteem—health systems need to address physical conditions in people with mental health problems
Commission pulmonary rehabilitation, as this is an extremely high-value intervention compared with other health interventions.
Attention to breathlessness in midlife has the potential to reduce sedentarism and the accumulation of multi-morbidities.

## Conclusion

Breathlessness is an important and challenging symptom. The Breathing SPACE approach should help to ensure that underlying diagnoses will not be missed, especially if there is more than one, and also that these are considered as patients are followed up over time. Focus attention on high-value interventions, in particular smoking cessation and exercise rehabilitation, to optimise the sustainable and equitable utilisation of finite health-care resources.
